# Experience in the management of lymphatic leakage using lipiodol lymphangiography and a temperature-sensitive liquid agent embolization

**DOI:** 10.3389/fradi.2026.1910524

**Published:** 2026-08-24

**Authors:** Bin Chen, Yuanhai Tu, Ximan Huang, Guiyuan Zhang, Keyu Tang, Haitao Dai, Yonghui Huang, Run Lin

**Affiliations:** 1 Department of Interventional Radiology, The First Affiliated Hospital, Sun Yat-Sen University, Guangzhou, China; 2 Department of Interventional Radiology, Huiya Hospital of the First Affiliated Hospital, Sun Yat-Sen University, Huizhou, China

**Keywords:** embolization, lipiodol, lymphangiography, lymphatic leakage, temperature-sensitive liquid agent

## Abstract

**Objective:**

Evaluate the safety and efficacy of a temperature-sensitive liquid embolic agent in the management of lymphatic leakage.

**Methods:**

Patients with lymphatic leakage who underwent interventional treatment in our center from September 2023 to April 2025 were retrospectively analyzed. The patient underwent percutaneous inguinal lymph node puncture, and iodized oil was used for lymphography. After lymphography, Chiba needle was used to puncture the breach under the guidance of DSA or CT, and temperature-sensitive liquid embolic agent (TempSLE) was used for direct injection embolization. The drainage volume or urine volume was mainly observed after procedure. The clinical success rate was defined as chest and abdominal drainage volume less than 100 mL/ day or urine chylous test negative for 3 consecutive days.

**Results:**

A total of 44 patients were included in this study. The main symptoms were chylous ascites in 26 cases, chylothorax in 12 cases, chylopericardium in 2 cases, and chyluria in 4 cases. Twenty-two patients had lymphatic leakage after surgical operation, mainly including Whipple procedures, spinal internal fixation and gynecological surgery. The remaining 22 cases were unexplained and spontaneous lymphatic leakage. Lipiodol lymphography was successfully performed in 44 patients, and embolization of lymphatic vessels using TempSLE were performed in 26 patients. The technique success rate was 100%, and clinical effective rate was 55.6% (lymphography alone group) and 84.6% (TempSLE group). No major complications were found in both groups.

**Conclusions:**

The application of temperature-sensitive liquid embolic agent (TempSLE) in the treatment of lymphatic leakage was safe and promising.

## Introduction

Lymphatic system dysfunction resulting in pathological fluid extravasation presents significant clinical challenges across multiple specialties. Postoperative or idiopathic lymphatic leakage predominantly manifests through three distinct clinical syndromes: chylothorax (thoracic duct incompetence), chylous ascites (abdominal lymphatic disruption), and chyluria (renal lympho-urinary fistula) (1). Current therapeutic algorithms progress from conservative management (dietary modification, somatostatin analogs) to invasive interventions, with contemporary guidelines prioritizing image-guided techniques over surgical ligation when anatomically feasible. Modern interventional lymphology employs a dual diagnostic-therapeutic paradigm combining lipiodol lymphangiography with targeted embolization. Three principal embolization strategies have been standardized: (1) Direct occlusion of lymphatic vessel wall defects, (2) Chemical ablation of segmental lymphatic networks through sclerotherapy, and (3) Retrograde nodal embolization to modulate lymphatic flow dynamics (2). N-butyl cyanoacrylate (NBCA) remains the current embolic agent of choice due to its rapid polymerization (<15 s) (3, 4), yet this very characteristic introduces critical limitations—including non-adaptable gelation kinetics, risk of non-target embolization from antegrade migration, and permanent mechanical alteration of delicate lymphatic architecture.

The temperature-sensitive liquid embolic agent (TempSLE, Beijing Guanhe Medical Technology Co. Ltd., Beijing, China), a novel thermosensitive polymer, is composed primarily of poly(N-isopropylacrylamide) copolymers. Upon entering the body, it undergoes a phase transition to form a gel in response to physiological temperature changes, thereby achieving vessel embolization.

In this study, we evaluated the safety and efficacy of Lipiodol lymphangiography and Embolization techniques using temperature-sensitive liquid embolic agent for the management of lymphatic leakage.

## Methods

### Patients

**Figure 1:**
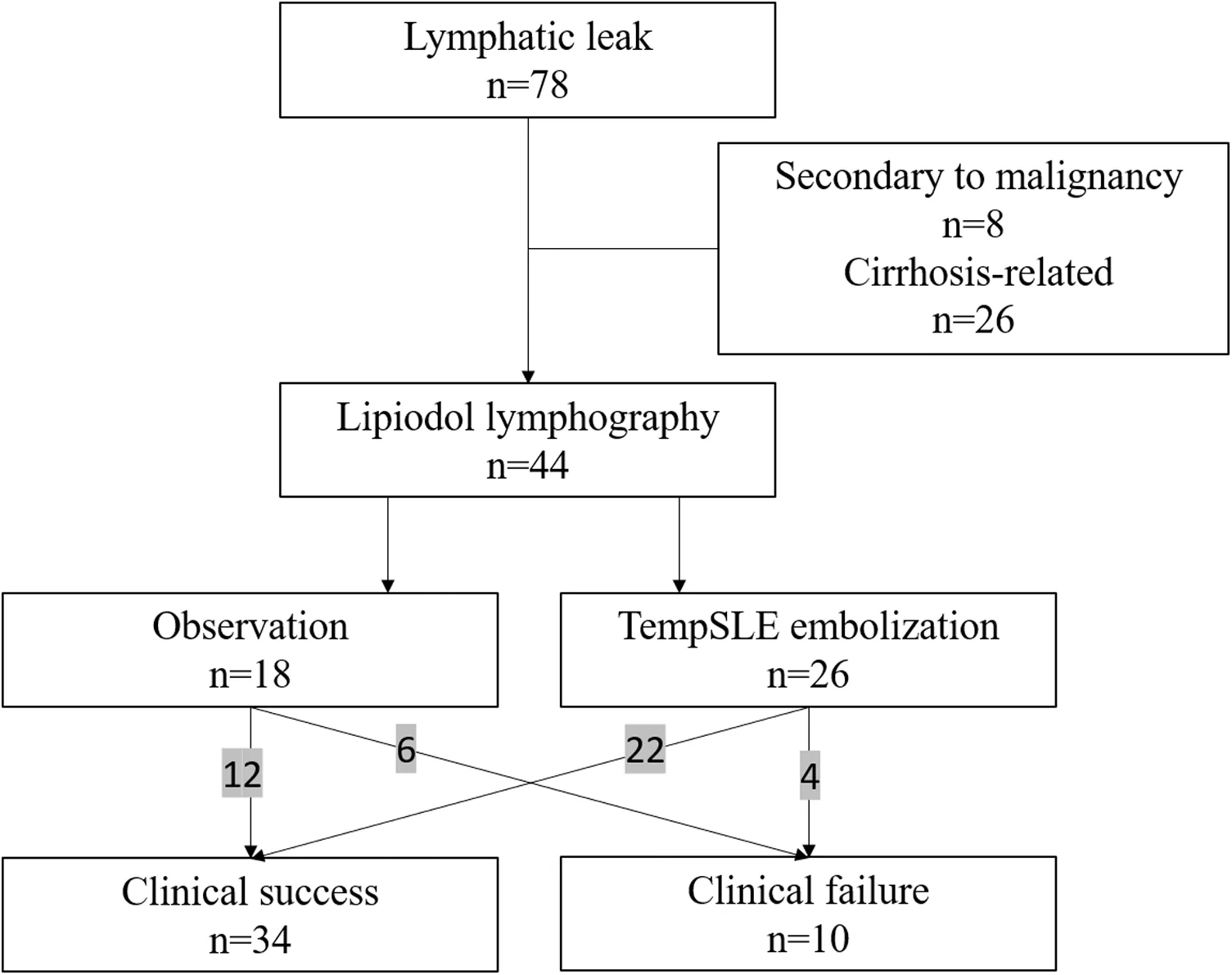
Patient Selection and Flowchart.

This retrospective study was approved by the Institutional Review Board (2025-1127-001). Given the retrospective nature of the study and the use of de-identified patient data, the requirement for informed consent was waived by the IRB. The study was conducted in accordance with the ethical standards of the Declaration of Helsinki and its later amendments. This study consecutively enrolled patients with lymphatic leakage who underwent diagnostic lymphography at our institution from September 2023 to April 2025, presenting with characteristic chylous effusions including chylothorax, chylous ascites, chylopericardium, or chyluria. Inclusion criteria as following: (1) age >18 years; (2) chylomicron analysis positive; (3) successful lymphography with iodized oil. Exclusion criteria encompassed: (1) chylous effusions secondary to malignancy; (2) cirrhosis-related chylous ascites; (3) cases with incomplete clinical follow-up data. The flowchart is shown in Figure 1.

### Pre-procedural conservative management

All patients initially received conservative management, including a low-fat diet with medium-chain triglycerides (MCT) before interventional treatment was considered. Somatostatin analogues were used at the discretion of the treating physician. TPN, MCT formula enteral nutrition, albumin supplementation, etc. would be performed as needed during the perioperative period.

## Procedures

### Lipiodol lymphangiography

**Figure 2:**
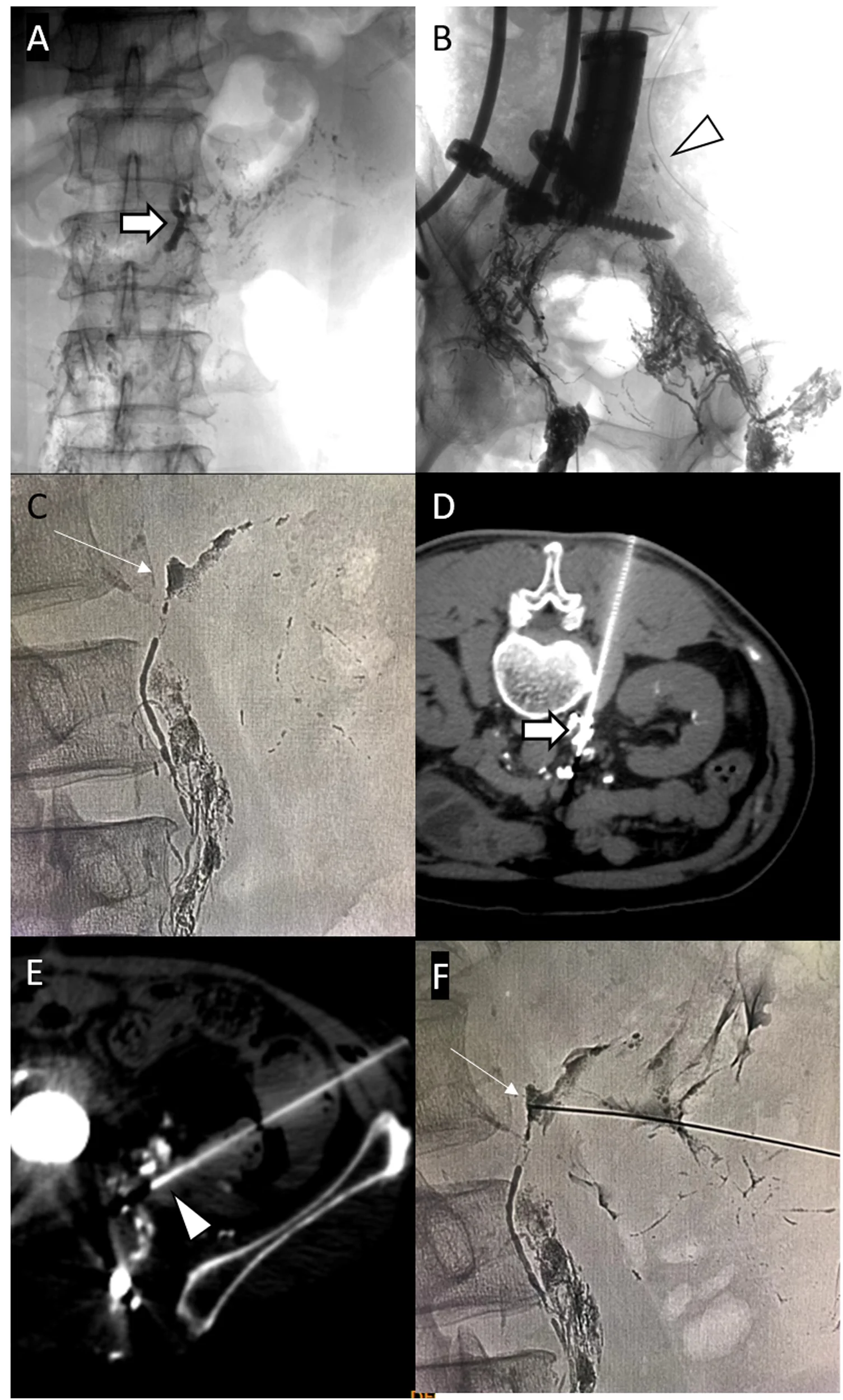
Typical interventional treatment images of lymphatic leakage. **(A)** A patient presenting with chyluria. Lipiodol lymphangiography demonstrates retrograde filling of Lipiodol into the left renal lymphatic system (arrow), confirming abnormal lymphatic-urinary communication. **(B)** A patient with chylous ascites following radical hysterectomy. Lipiodol lymphangiography reveals Lipiodol extravasation from the presacral lymphatic plexus (triangle arrow) into the peritoneal cavity, with subsequent accumulation within an abdominal drainage catheter (asterisk). **(C)** A patient with chylous ascites post-Whipple procedure (pancreaticoduodenectomy). Lipiodol lymphangiography identifies active leakage at the left L3 lumbar lymphatic trunk (thin arrow), with Lipiodol pooling in the peritoneal cavity. **(D)** CT-guided embolization of lymphatic vessels draining into the left renal region for (A). A mixture of Lipiodol and temperature-sensitive gel (TempSLE) was injected to occlude the pathological lymphatic channels (arrow), achieving complete resolution of chyluria. **(E)** CT-guided direct puncture of the lymphatic leakage site adjacent to the drainage catheter. TempSLE was injected to achieve targeted embolization (triangle arrow) for (B), resulting in immediate cessation of chylous fluid output. **(F)** Fluoroscopy-guided direct puncture of the leaking lumbar lymphatic vessel. A combination of TempSLE and Lipiodol was administered to embolize the leakage site (thin arrow), effectively sealing the defect and resolving ascites for (C).

The patient is positioned supine, and the bilateral inguinal regions along with the abdominal area undergo standardized aseptic preparation and draping. Under real-time ultrasound guidance, percutaneous puncture of the lymphatic hilum within unilateral or bilateral inguinal lymph node groups is achieved using a 20-gauge needle. Iodized oil contrast medium (Lipiodol® Ultra-Fluide, Guerbet) is administered via controlled slow injection until progressive opacification of regional lymphatic vessels is radiographically demonstrated. Subsequently, contrast administration is maintained at a constant rate of 0.1 mL/min under fluoroscopic monitoring until definitive visualization of thoracic duct termination at the venous angle is attained. Multiplanar fluoroscopic evaluation is systematically performed to detect potential contrast extravasation indicative of lymphatic leakage or morphological abnormalities suggestive of pseudoaneurysm formation within the lymphatic network as Figures 2A–C.

### Embolization

If a clear leakage point was identified, the patient proceeded to targeted TempSLE embolization. If no clear leakage point was detected on lymphangiography, the patient received Lipiodol lymphangiography alone, as there was no target for embolization. Following identification of the lymphatic leakage site, percutaneous access to the affected lymphatic vessel or perilesional collateral vessel is meticulously established under CT or fluoroscopy guidance using a 21-gauge Chiba needle as Figures 2D,E or real-time fluoroscopic as Figure 2F. Upon confirmation of proper needle positioning within the targeted lumen through multiplanar imaging verification, controlled, stepwise manner injection of the temperature-sensitive liquid embolic agent (TempSLE,) was performed under continuous image monitoring without dilution. The embolization endpoint was defined radiologically as complete cessation of iodized oil contrast extravasation, confirmed by sustained fluoroscopic observation across multiple respiratory phases.

### Complication

All periprocedural adverse events were systematically recorded and subsequently categorized *post hoc* as minor or major under strict adherence to the Society of Interventional Radiology Standards of Practice Committee (5).

### Technical and clinical success

Technical success was objectively defined as the completion of both diagnostic lymphographic mapping using iodized oil contrast and subsequent embolization of iodized oil. Clinical success required biochemical and volumetric criteria: biochemical confirmation of chylous fluid clearance (documented conversion from positive to negative in chylomicron analysis) combined with sustained reduction of daily drainage output to <100 mL/day over three consecutive measurements.

## Results

A total of 44 patients were included in the study, divided into two groups: Lipiodol lymphangiography alone (*n* = 18) and Lipiodol lymphangiography combined with embolization (*n* = 26). Baseline characteristics and outcomes are summarized in Table 1. The mean age of patients was comparable between the two groups (52.8 ± 12.4 years vs. 51.8 ± 15.9 years, *p* = 0.875). A higher proportion of male patients was observed in the embolization group (77.0% vs. 33.3%). The primary indication for the procedure was chylous ascites, accounting for 55.6% (10/18) in the lymphangiography group and 61.5% (16/26) in the combined embolization group. Other indications included chylothorax (33.3% vs. 23.1%) and chyluria (0% vs. 7.7%). The duration of symptoms, dose of lipiodol, and technical success were similar in both groups. Clinical success rates were 55.6% (10/18) in the lymphangiography group and 84.6% (22/26) in the embolization group (*p* = 0.075). Although there was no significant difference, the clinical success rate of the embolization group was higher than that of the lymphangiography group in the trend.

**Table 1:** Patient characteristics and outcomes.

Characteristic	Lipiodol lymphangiography *n* = 18	Lipiodol lymphangiography combined embolization *n* = 26	*p*-value
Age (year)	52.8 ± 12.4	51.8 ± 15.9	0.875
Gender (male)	6 (33.3%)	20 (76.9%)	–
Indication for the procedure			–
Chylothorax	6 (33.3%)	6 (23.1%)	
Chylous ascites	10 (55.6%)	16 (61.5%)	
Chyluria	0	2 (7.7%)	
other	2 (11.1%)	2 (7.7%)	
Duration of symptom (month)	2.6 ± 2.4	1.3 ± 0.6	0.155
Previous Treatment			–
Surgery	6 (33.3%)	16 (61.5%)	
No obvious causes	12 (66.7%)	10 (38.5%)	
Number of lymphangiography	1.11	1.23	–
Dose of Lipiodol (mL)	21.4 ± 5.3	23.4 ± 7.2	0.501
Liquid embolic agent (mL)	0	3.1 ± 1.5	–
Procedural duration (min)	137.5 ± 31.2	128.1 ± 54.7	0.473
Technique success	100%	100%	–
Clinical success	10 (55.6%)	22 (84.6%)	0.075
Clinical resolution time (day)	13.6 ± 5.7	6.2 ± 6.5	0.004

### Follow-up and recurrence

The median clinical follow-up was 11 months (range, 3–19 months) from the date of intervention. The average time from intervention to clinical resolution (defined as drainage <100 mL/day for 3 consecutive days or a negative chylous test) was 13.6 ± 5.7 days in the lymphangiography-alone group and 6.2 ± 6.5 days in the TempSLE embolization group. Among patients who achieved clinical success, the median time to resolution was 6 days (range, 1–30 days). Among patients who achieved clinical success, none of patients experienced recurrence of lymphatic leakage during the follow-up period.

### Complication

The adverse events were classified according to the Society of Interventional Radiology Standards of Practice Committee (5). As shown in Table 2, no major complications related to lymphangiography or embolization were reported. The most common minor complication was pain after puncture for embolization, which resolved without intervention. During the available clinical follow-up period, no cases of clinically apparent lymphedema were reported among the treated patients.

**Table 2:** Major and minor complication.

Complication	Lymphangiography and/or embolization
Major complications	
Bleeding	0
pulmonary infarction	0
Minor complications	
Pain of puncture point	8
limb lymphedema	0
bleeding	0
fever	0

## Discussion

Postoperative lymphatic fistula is a severe complication after thoracic, abdominal, or pelvic surgeries resulting in high mortality. Loss of fluid, triglyceride, lymphocyte and immunoglobulin, from the leakage of lymphatic vessels may lead to dehydration, nutritional deficiency and immunologic dysfunction (6). Lipiodol lymphangiography not only allows for precise localization of lymphatic leakage sites—utilizing lymphatic permeability and retention properties via imaging—but may also provide therapeutic effects through physical occlusion or the induction of localized inflammatory responses (7, 8). However, approximately 50% of patients required additional interventions following lymphangiography alone, indicating limitations in its therapeutic efficacy (4, 9).

In this study, lymphatic leakage (lymphatic fistula) was observed in 22 out of 44 patients following surgical procedures, including Whipple procedures (pancreaticoduodenectomy), as well as gynecologic, orthopedic, and urologic surgeries. These findings suggest that iatrogenic injury to the lymphatic system—primarily caused by intraoperative lymph node dissection or inadvertent damage to lymphatic vessels during surgical manipulation—is the predominant cause of lymphatic leakage in this cohort. A part of postoperative lymphatic leaks resolves spontaneously with conservative management, while others achieve closure with adjunctive therapies such as fasting, somatostatin analogs, or nutritional optimization. However, in cases that are refractory to aggressive medical treatment, particularly those presenting with persistent chylous effusions, percutaneous lymphangiography with lymph node puncture becomes a critical diagnostic and therapeutic intervention. This approach allows for precise localization of leakage sites and facilitates targeted embolization or sclerotherapy, thereby addressing the underlying pathology when conventional methods fail.

Percutaneous lymph node puncture with Lipiodol lymphangiography serves as the foundational step for interventional therapies targeting the lymphatic system. This imaging modality enables visualization of the drainage pathways of inguinal lymph nodes and the identification of lymphatic leakage sites. Lipiodol, through its dual mechanisms of local inflammatory response and embolic effects within lymphatic vessels, promotes spontaneous closure of leakage points, with reported efficacy rates ranging from 20% to 85% across studies (2, 3, 8). However, due to the variability in therapeutic outcomes, Lipiodol lymphangiography must be combined with adjunctive embolization techniques, such as direct leakage site embolization or occlusion of feeding lymphatic vessels or upstream lymph nodes, to achieve consistent clinical success. Common embolic agents include N-butyl cyanoacrylate (NBCA) glue and metallic coils (10). In a seminal study, the combination of Lipiodol lymphangiography with NBCA glue embolization achieved a clinical success rate of 85% (3). Nevertheless, NBCA glue has been reported to have limitations (3), including procedural pain during injection and potential complications such as tissue necrosis or vascular injury if accidental non-target embolization occurs.

TempSLE is a novel temperature-sensitive liquid embolic agent that was introduced in 2020. Its material is a copolymer of N-isopropylacrylamide and N-propylacrylamide cross-linked by N, N-methylene diacrylamide. TempSLE belongs to the class of temperature-sensitive hydrogels known as poly(N-isopropylacrylamide) (pNIPAAm), which undergo a sol-to-gel transition in response to temperature changes (11). The transition temperature is approximately 32 °C, and at temperatures above this threshold, it transforms into a gel state. The incorporation of TempSLE into interventional treatment for lymphatic leakage offers unique advantages. Unlike commonly used liquid embolic agents in clinical practice, such as iodized oil, which remain in liquid form at body temperature (around 37 °C), TempSLE transforms into a solid gel at this temperature, providing better embolization efficacy. During the injection of N-butyl cyanoacrylate (NBCA) glue, patients may experience significant pain. Additionally, since NBCA lacks inherent radiopacity under x-ray imaging, it must be mixed with Lipiodol to visualize the embolization range. In the present study, TempSLE demonstrated excellent x-ray visibility, allowing precise real-time monitoring of the embolic distribution. Furthermore, its administration was associated with no significant discomfort in patients, suggesting improved tolerability compared to traditional NBCA-Lipiodol mixtures.

In this study, the injection protocols for TempSLE varied based on the procedural guidance modalities used. The clinical success rate of using TempSLE for embolization or sclerosis treatment reached 84.6%, which was close to the using NBCA glue (3). In 5 cases, a direct fluoroscopy-guided puncture of the leakage site was performed using a percutaneous transhepatic cholangiography (PTC) needle, followed by immediate injection of TempSLE after Lipiodol lymphangiography. Combination with CT-guided embolization was a promising treatment option for the management of refractory lymphatic leakage (12). In the remaining 8 cases, CT-guided embolization was employed; the Chiba needle was advanced into the lymphatic vessels or thoracic duct, previously identified as the leakage source via Lipiodol lymphangiography, to deliver TempSLE. Post-embolization, lymphatic leakage ceased or markedly decreased in all cases, confirming the therapeutic value of precise source-level occlusion.

Recent large series of thoracic duct embolization (TDE) have reported clinical success rates ranging from 70% to 90% in patients with chylothorax and chylous ascites (13, 14). Our overall clinical success rate of 84.6% (22/26) with TempSLE embolization compares favorably with these benchmarks, despite the heterogeneity of our cohort (including postoperative and idiopathic leaks). For postoperative lymphatic leaks specifically, Hur et al (3). achieved a 94% clinical success rate using NBCA glue embolization in 27 patients, while Pan et al. (2) and Kaminski et al. (4) reported success rates of 76%–89% with combined lipiodol lymphangiography and adjunctive embolization. Our results are consistent with these prior reports and suggest that TempSLE, with its unique thermosensitive properties and inherent radiopacity, may offer a viable alternative to conventional agents, particularly in cases where NBCA-related pain or non-target embolization is a concern. However, direct comparisons are limited by differences in patient selection, leakage etiology, and embolization techniques, and our follow-up duration was shorter than in some of the cited series.

This study has several limitations. First, the sample size was relatively small. The heterogeneous nature of the cohort—which includes patients with chylothorax, chylous ascites, chylopericardium, and chyluria, as well as both postoperative and spontaneous etiologies—precludes meaningful subgroup analyses given the current sample size. Second, the current interventional therapy for lymphatic leakage requires experienced operators, and the treatment protocols continue to be optimized as operators gain more experience. Additionally, improvements are needed in the injection ratio and speed of TempSLE during the procedure, as well as in the related procedural equipment, to enhance the precision of interventional therapy for lymphatic leakage. Short-term follow-up may not capture delayed complications such as lymphedema or leak recurrence, and that future prospective studies with extended surveillance are warranted.

## Conclusion

The application of a temperature-sensitive liquid agent provides a safe, promising trend, minimally invasive solution for the treatment of lymphatic leakage. these are preliminary findings requiring validation in larger, prospective, preferably multi-center randomized studies.

## Data Availability

The raw data supporting the conclusions of this article will be made available by the authors, without undue reservation.
